# In silico analysis of radiation-induced double-strand breaks by internal ex vivo irradiation of lymphocytes for 45 alpha- and beta/gamma-emitting radionuclides

**DOI:** 10.1186/s13550-025-01214-w

**Published:** 2025-03-10

**Authors:** Maikol Salas-Ramirez, Michael Lassmann, Uta Eberlein

**Affiliations:** Department of Nuclear Medicine, University Hospital Würzburg, Würzburg, Germany

**Keywords:** Monte Carlo simulation, Geant4-DNA, GATE, Blood dosimetry, DNA damage

## Abstract

**Background:**

The aim of this study is to evaluate the induction of DNA damage by 45 radionuclides, including those used in medical applications and others relevant to radiation protection. The research focuses on understanding the differential effects of irradiating lymphocytes with beta/gamma- and alpha-emitting radionuclides using Monte Carlo simulations. A validated Monte Carlo simulation model was used to assess radiation-induced DNA damage in lymphocytes. The model integrates GATE for macroscopic radiation transport and Geant4-DNA for microscopic simulations at the cellular level. For the study, 45 radionuclides were selected and their S-values and DNA double-strand break (DSB) induction were investigated. For beta- and gamma-emitting radionuclides, DSBs per cell per mGy were quantified, while for alpha-emitters, alpha tracks per cell per mGy, DSBs per cell per mGy, and DSBs per micrometer of alpha track were calculated.

**Result:**

For beta/gamma emitters, the lowest number of DSBs was observed with ^125^I at 0.006 ± 0.003 DSBs·cell⁻¹·mGy⁻¹, while ^99m^Tc had the highest at approximately 0.015 ± 0.005 DSBs·cell⁻¹·mGy⁻¹. The S-value for lymphocyte nuclei ranked from 0.91 ± 0.14 mGy∙h⁻¹∙MBq⁻¹ (^63^Ni) and 1.06 ± 0.15 mGy∙h⁻¹∙MBq⁻¹ (^125^I) to 61.83 ± 1.17 mGy∙h⁻¹∙MBq⁻¹ (^90^Sr). For alpha-emitting radionuclides, ^213^Bi produced 0.0677 ± 0.0005 DSB·cell⁻¹·mGy⁻¹ while ^232^Th yielded 0.0914 ± 0.0004 DSB·cell⁻¹·mGy⁻¹. The DSB linear density for alpha tracks ranged from 7.4 ± 0.1 DSBs/µm for ^252^Cf to 16.8 ± 0.1 DSBs/µm for ^232^Th. The S-values for lymphocyte nuclei for alpha emitters varied, from ^232^Th (0.29 ± 0.21 Gy∙h⁻¹∙MBq⁻¹) to ^227^Th having the highest at 2.22 ± 0.16 Gy∙h⁻¹∙MBq⁻¹, due to cumulative energy deposition.

**Conclusions:**

Differences were observed in DNA damage induced by beta/gamma- and alpha-emitting radionuclides. High-energy beta emitters induced DSBs similarly to gamma emitters, but with greater fluctuations in low-energy beta and gamma emitters due to heterogeneous energy deposition and varying interaction probabilities at the cellular level. This study highlights that long half-life alpha-emitting radionuclides may cause more extensive DNA damage due to their higher LET. This work provides a comprehensive S-values database for future experimental studies on radiation-induced DNA damage in lymphocytes.

**Supplementary Information:**

The online version contains supplementary material available at 10.1186/s13550-025-01214-w.

## Introduction

After incorporation of radionuclides into the body, these radionuclides are often distributed through the bloodstream, where they can cause DNA damage in peripheral blood mononuclear cells (PBMCs). The response of DNA to damage induced by ionizing radiation (IR) can be characterized by the formation of γ-H2AX and 53BP1 radiation-induced foci (RIF), which are indicative of double-strand breaks (DSBs) [1]. This response can be detected using biodosimetric methods, particularly through the DNA damage assay. In recent years, the γ-H2AX + 53BP1 assay has become widely used for quantifying radiation-induced DSBs by counting microscopic foci containing these biomarkers (e.g [1, 2]). Especially for PBMCs, the method is now well established for internal irradiation in nuclear medicine [3–6]. International efforts are on-going to characterize and to standardize the results of this assay further [7].

Monte Carlo (MC) track structure simulations have significantly enhanced our understanding of how IR interacts with DNA, specifically in simulating the induction of single- and double-strand breaks (SSBs and DSBs) [8–10]. The main advantage of Monte Carlo track structure simulations is registering the spatial distribution of interactions of IR with DNA and quantifying the amount of deposited energy in each interaction [11]. Therefore, the Monte-Carlo simulated DNA DSB induction is a potential counterpart to the γ-H2AX + 53BP1 assay [12].

In a recent study, we validated a Monte Carlo track structure model against experimental data obtained from internal ex vivo irradiation of PBMCs with several alpha-, beta- and gamma-emitting radionuclides used in nuclear medicine for absorbed doses of about 50 mGy [12]. The MC-calculation was based on a clustering algorithm that quantifies the number of DSBs produced in the PBMCs’ nucleus by using Geant4-DNA [13–17] codes at the microscopic level. Moreover, a cluster analysis was implemented to establish the first data set of the number of clusters vs. cluster size (number of strand breaks) for internal ex vivo irradiation with radionuclides used in nuclear medicine.

Monte Carlo simulations present distinct advantages, particularly in cases where experimental studies are either impractical or inaccessible due to safety concerns or logistical challenges. This study employs an in silico approach to analyze radiation-induced DNA damage across a comprehensive range of radionuclides. These include those that are used in medical applications as well as those produced in nuclear power plants, research reactors and radionuclide production facilities. Most of the radionuclides are studied in the context of radiation protection for workers under monitoring programs and of potential nuclear disaster scenarios.

On the basis of the recently established model [12], the primary objective of this study is, therefore, to assess DNA damage induction for 45 alpha and beta/gamma emitters, for which experimental data are not easily available. Using Monte Carlo simulations, this research aims to fill gaps in our understanding of radiobiology and provide critical data for radiation protection, particularly in occupational settings, and for optimizing therapeutic applications involving ionizing radiation.

## Materials and methods

This study employs a validated Monte Carlo simulation model [12] to assess radiation-induced DNA damage in lymphocytes. The simulation process integrates both macroscopic and microscopic scales, using GATE (Geant4 Application for Tomographic Emission) [13, 14] for macroscopic radiation transport and Geant4-DNA [15, 16, 18] for microscopic simulation at cellular level.

### Radionuclide selection

A total of 45 radionuclides (^14^C, ^32^P, ^33^P, ^35^S, ^36^Cl, ^59^Fe, ^54^Mn, ^60^Co, ^63^Ni, ^67^Cu, ^75^Se, ^89^Sr, ^90^Sr, ^90^Y, ^99m^Tc, ^123^I, ^125^I, ^131^I, ^134^Cs, ^137^Cs, ^153^Sm, ^161^Tb, ^177^Lu, ^188^Re, ^192^Ir, ^210^Po, ^211^At, ^212^Bi, ^213^Bi, ^220^Rn, ^222^Rn, ^223^Ra, ^224^Ra, ^225^Ac, ^226^Ra, ^227^Th, ^228^Ra, ^228^Th, ^232^Th, ^237^Np, ^238^Pu, ^239^Pu, ^241^Am, ^244^Cm, ^252^Cf) were explored. These radionuclides were selected based on their listing in official radiation protection documents for monitoring occupational intakes of radionuclides [19], risk analyses reports [20], and reviews on trends in radiopharmaceutical development [21–23].

Radionuclides such as ^90^Y, ^99m^Tc, ^123^I, ^131^I, ^177^Lu, ^223^Ra, and ^225^Ac, which have been previously studied and validated [12], were included to provide a comprehensive view. In our previous study we provided the absorbed dose coefficients for 1 ml for 1 h internal ex vivo irradiation of peripheral blood (d_Blood_) and for the lymphocyte nuclei (d_Lymph_), while in this study the S-values for peripheral blood (S_Blood←Blood_) and for the lymphocyte nuclei (S_Lymph←Blood_) are provided. For actinides (^232^Th, ^237^Np, ^239^Pu, ^241^Am, ^244^Cm, ^252^Cf), the decay chain was simulated in steps, stopping at long half-life daughters (over 15 days) because achieving secular equilibrium may not be feasible in potential experimental settings. Specific cases include ^222^Rn (cut at ^210^Pb), ^226^Ra (cut at ^210^Po), ^232^Th (cut at ^228^Ra), ^237^Np (cut at ^233^Pa), ^238^Pu (cut at ^234^U), ^239^Pu (cut at ^235^U), ^241^Am (cut at ^237^Np), ^244^Cm (cut at ^240^Pu), and ^252^Cf (cut at ^248^Cm). Additionally, ^228^Ra decay chain was stopped at ^228^Th, while ^211^At simulations excluded ^207^Bi due to its long half-life (32.9 years). Similarly, the chain for ^225^Ac and ^213^Bi was stopped at ^209^Bi (half-life: 2.01 × 10^19^ years). For the remaining alpha-emitting radionuclides the entire decay chain was simulated. ^212^Pb was not explicitly selected because it undergoes beta decay to ^212^Bi, which subsequently decays via two branches, both involving beta and alpha emissions. Given that one of the aims is to investigate alpha particle-induced DNA damage, ^212^Bi was included in the analysis as the first relevant alpha-emitting radionuclide in the decay chain.

Positron-emitting radionuclides (e.g., ^18^F, ^68^Ga, ^124^I) were not included due to the absence of positron interaction physics processes in Geant4-DNA.

### Simulation geometry and experimental setup

The simulation geometry replicated an 8 mL vial containing a water-equivalent radioactive solution. Within this volume, 1000 lymphocyte spheres were randomly distributed.

Each lymphocyte was modeled as a sphere with a radius of 3.75 μm, encapsulating a nucleus modeled as a concentric sphere with a radius of 3.1 μm. This dimension corresponds to the mean radius for lymphocytes measured using scanning flow cytometry [24]. Furthermore, at the macroscopic level, all incident particles are scored in a phase-space attached to each lymphocyte sphere. In the context of this study, phase-space is defined as a database of particles emitted from the blood volume and interacting with lymphocytes (scoring surface). Each particle is scored only once during its passage. This database includes detailed particle properties such as energy, type, position, direction, production volume, and the process responsible for particle generation (e.g., decay, photoelectric effect, Compton scattering).

In this study, the number of lymphocytes in the 8 mL vial was set to 1000, representing a compromise between computational feasibility and statistical accuracy. The primary objective was to maintain a relative uncertainty of the deposited energy below 5% in the microscopic simulation (Geant4-DNA). Increasing the number of lymphocytes would reduce the number of simulated particles per cell in the macroscopic simulation (GATE) without providing additional information. The random distribution of lymphocytes ensures phase-space scoring across the entire vial, while summing all phase-spaces in the microscopic simulation into one single phase-space enhances statistical accuracy. Given the number of simulations and constraints on computer memory (random-access memory), extending the simulation time was preferred over increasing the number of lymphocytes. Further description of the simulation settings are available in Salas-Ramirez et al. [12].

### Source, physics processes, and number of simulated particles

As described in our previous study [12], the source was simulated at the macroscopic level using the GATE ion source description. When the ion source is used a simulated particle corresponds to a nuclear transformation (one nuclear decay). Furthermore, the physics list QGSP_BIC_EMZ (hadrons and electromagnetic processes) for all radionuclides was used, with the exceptions of ^244^Cm and ^252^Cf, which employed QGSP_BIC_HP_EMZ (hadrons, neutrons below 20 MeV and electromagnetic processes) to simulate and transport all particles produced throughout the decay scheme. While neutrons were transported in the macroscopic simulation, they were removed from the phase-space in the microscopic simulation, as the focus for alpha-emitting radionuclides was alpha tracks. Moreover, Geant4-DNA lacks interaction physics processes to simulate neutrons.

The number of particles simulated in GATE was adjusted to ensure that the relative uncertainty of the deposited energy in the lymphocyte nucleus remained below 5% in the Geant4-DNA simulation. At the macroscopic level, simulation times range from four to eight days for beta- and gamma-emitters and approximately four weeks for alpha-emitters when performed on a single CPU. At the microscopic level, beta- and gamma-emitters require no more than an hour, while alpha-emitters take approximately one day.

Radionuclides such as ^3^H, ^51^Cr, ^57^Co, ^58^Co, ^201^Tl, and ^149^Tb were initially simulated but subsequently excluded. For ^3^H, ^51^Cr, ^57^Co, ^58^Co, and ^201^Tl, achieving an uncertainty below 5% required the simulation of more than 8 × 10⁹ particles, making their inclusion difficult due to the long computational time required. ^149^Tb (half-life: 4.12 h) is a partial alpha-emitter that decays via two branches: 16.7% of decays proceed through alpha emission to ¹⁴⁵Eu (half-life: 5.93 days, mean energy: 3.967 MeV), while 83.3% undergo electron capture to ¹⁴⁹Gd (half-life: 9.28 days) [25, 26]. All subsequent daughters in both decay chains decay mainly via electron capture. Therefore, the decay chains of ^149^Tb were truncated at ^145^Eu and ^149^Gd as a simulation of 4 × 10^9^ particles (macroscopic level) registered 89 alphas in the phase-space (microscopic level). This number of particles is insufficient to reach an uncertainty of less than 5%, and further simulation at the macroscopic level was impractical due to the long computational time. Variance reduction techniques were not considered, as they may lead to incorrect or complex uncertainty estimation or introduce dependencies between particles (e.g., phase-space recycling). The number for simulated nuclear transitions for each radionuclide is provided in Table 1 of supplementary material.

At the microscopic level, the phase-space was used as source. For beta emitting radionuclides, the anti-neutrino particles were removed from the phase-space to reduce the size of the database. In the case of alpha emitting radionuclide only the alpha particles were transported.

### Clustering algorithm and DNA damage metrics

The clustering algorithm implemented in Geant4-DNA, specifically the DBSCAN (Density-Based Spatial Clustering of Applications with Noise) algorithm [27], is used to quantify DNA damage in the form of double-strand breaks (DSBs). The algorithm identifies clusters of energy deposition events within the DNA’s sensitive volume, which was modeled as a 7% of the lymphocyte nucleus. This 7% value derived from the physical properties of DNA and its distribution within the lymphocyte nucleus. Specifically, the DNA volume is estimated to be 8.3 μm³ and modeled as a cylinder with a radius of 1.1 × 10⁻⁹ m [28] and a length of 2.2 m. This corresponds to the DNA density of 0.34 × 10⁻⁹ m per base pair [28] multiplied by 6.4 × 10⁹ base pairs in the human genome [29]. Conversely, the lymphocyte nucleus is modeled as a sphere with a radius of 3.1 × 10⁻⁶ m, resulting in a total volume of 124.8 μm³. The ratio of the DNA volume to the nucleus volume is approximately 0.07 (7%; 8.3 μm³ / 124.8 μm³) [12]. This 7% reflects the proportion of the nucleus occupied by DNA. This approach has been previously validated and described in existing literature [12]. The clustering algorithm groups single-strand breaks (SSBs) into double-strand breaks (DSBs) based on a damage probability, which starts at 0% for energies below 5 eV and increases linearly to 100% at 37.5 eV and above. A DSB is defined as two SSBs separated by less than 10 base pairs (∼3.4 nm) [27]. Furthermore, the clustering algorithm quantifies induced DNA damage and does not account for DNA repair. In total five simulations, each with a different seed, were performed to calculate the standard deviation of the number of DSBs and alpha tracks.

The following four metrics were calculated in this study:

#### DSBs per cell per mGy

For beta-emitting radionuclides, the average number of DSBs induced in the lymphocyte nucleus per unit of absorbed dose in whole blood was calculated. This metric is compared with the experimental results of Eberlein et al. [3] and Schumann [30]. Both studies provide data for clinically relevant radionuclides.

#### Alpha tracks per cell per mGy

For alpha-emitting radionuclides, the number of alpha particle tracks in the lymphocyte nucleus per unit of absorbed dose in whole blood was calculated. This metric is compared with the experimental results of Göring et al. [4], which quantify the number of alpha tracks produced by ^223^Ra. All results for alpha-emitting radionuclides are expressed in units of α-tracks·cell⁻¹·mGy⁻¹.

#### DSBs per cell per mGy

The total number of DSBs induced in the lymphocyte nucleus by alpha particles was normalized by the number of cells and the absorbed dose in whole blood to obtain the DSB·cell⁻¹·mGy⁻¹ metric. These values were compared with another Monte Carlo simulation [31], which is also based on Geant4-DNA but employs a different DNA damage scoring technique and a distinct sensitive volume (a fractal-based DNA chain structure of approximately 6.4 Gbp in length) compared to that used in this study.

#### DSBs per micrometer

For alpha-emitting radionuclides, the density of DSBs along the alpha particle tracks in the lymphocyte nucleus was calculated. This parameter is of particular relevance in experimental studies that utilize high-resolution microscopic techniques to estimate the number of DSBs in an alpha track [32].

### Calculation of S-values

In the previous study [12], the absorbed dose coefficients for lymphocyte nuclei (d_Lymph_) and whole blood absorbed dose coefficients (d_Blood_) for one hour of irradiation were calculated. In this study the S-values for lymphocyte nuclei (S_Lymph←Blood_), where lymphocyte nuclei are the target and blood (extracellular space) is the source, and for whole blood (S_Blood←Blood_), were calculated. The calculation of S-values, expressed in units of Gy·MBq^− 1^·h^− 1^, enables for the estimation of absorbed dose across a range of activity and irradiation time combinations. This approach offers greater flexibility in the design and analysis of ex vivo irradiation experiments. For evaluation of the calculation method the S_Lymph←Blood_-value for ^90^Sr and ^125^I were compared with the S-value for an 8 cm^3^ sphere (same volume as the whole blood volume in this study) from the IDAC-Dose [33] spheres model.

## Results

### Beta- and Gamma-emitting radionuclides

#### DSBs quantification

**Fig. 1:**
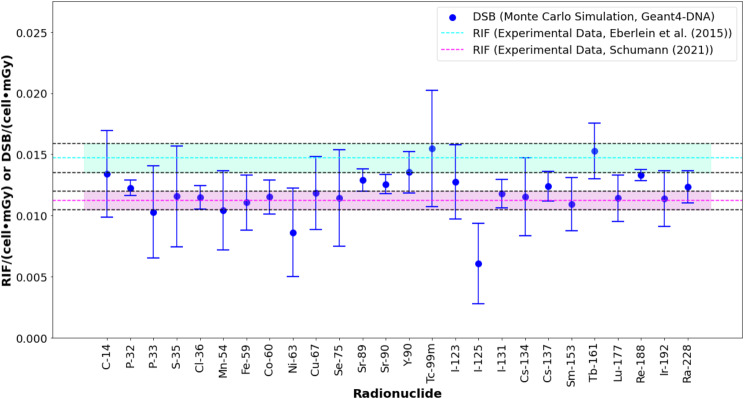
Number of DSBs per cell per mGy for beta- and gamma-emitting radionuclides. Error bars consider a coverage factor (k) of 2. ^90^Y, ^99m^Tc, ^123^I, ^131^I, and ^177^Lu were previously studied and validated in [12]

The mean number of DSBs per cell per mGy, illustrated in Fig. 1, aligns well with the experimental data from Eberlein et al. [3] and Schumann [30]. The highest DSB yield was observed with ^99m^Tc at 0.015 ± 0.005 DSBs·cell⁻¹·mGy⁻¹, though this radionuclide also exhibited the highest standard deviation. ^125^I had the lowest value at approximately 0.006 ± 0.003 DSBs·cell⁻¹·mGy⁻¹. Most radionuclides showed values close to the experimental data, except for ^125^I. Numerical results are detailed in supplemental Table 2.

#### S-values

**Fig. 2:**
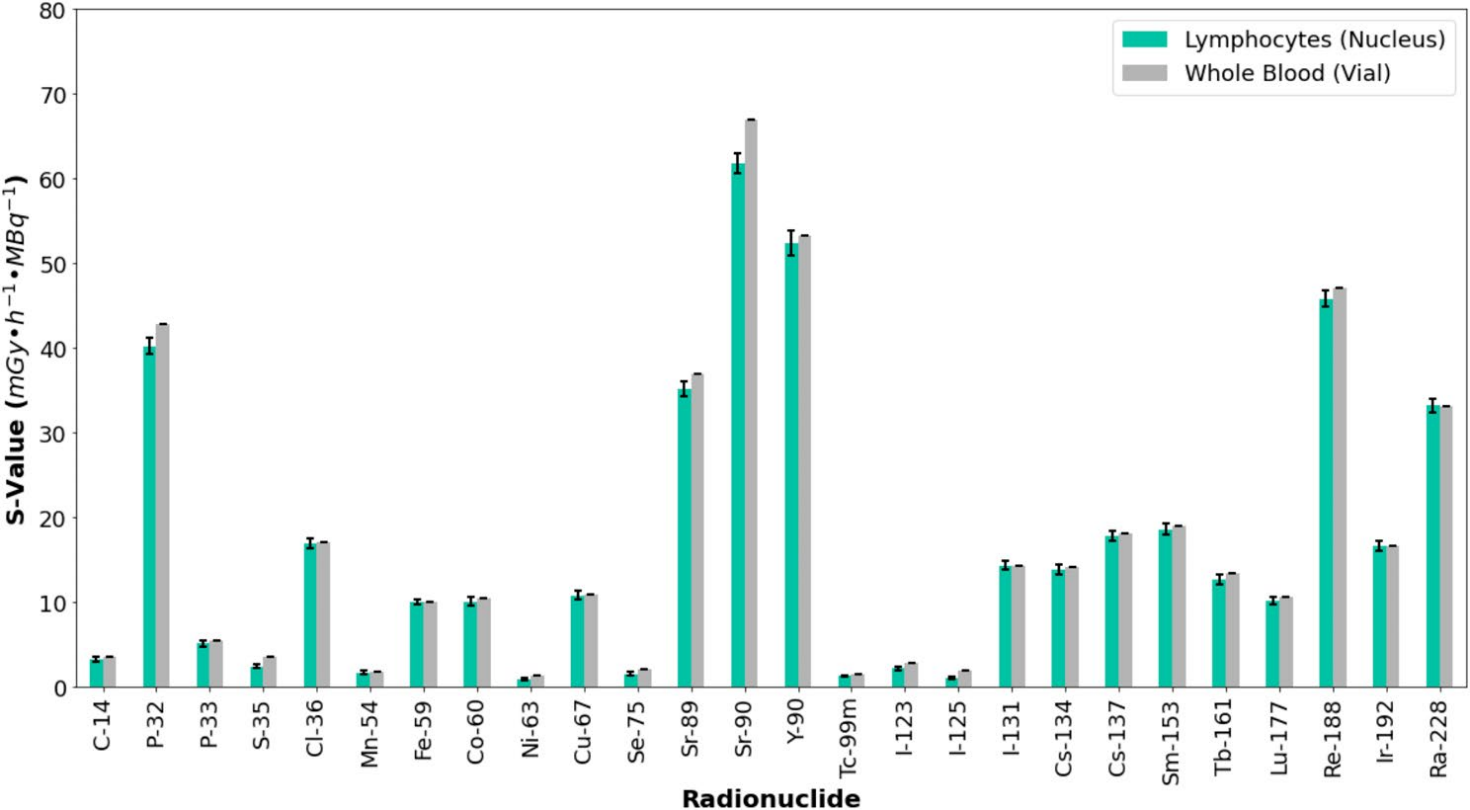
S-values for beta- and gamma-emitting radionuclides: Comparison between lymphocyte nuclei (S_Lymph←Blood_) and whole blood (S_Blood←Blood_). Error bars consider a coverage factor (k) of 2

As shown in Fig. 2, the maximum S-value was observed for ^90^Sr, with S_Lymph←Blood_ = 61.83 ± 1.17 mGy∙h^− 1^∙MBq^− 1^ and S_Blood←Blood_ = 66.92 ± 1E-3 mGy∙h^− 1^∙MBq^− 1^, consistent with the radionuclide’s high beta energy emissions and subsequent decay into ^90^Sr through ^90^Y. A comparison with an 8 cm^3^ sphere (same volume as the whole blood volume in this study) in IDAC-Dose [33], which provides a value of 13.76 mGy∙h^− 1^∙MBq^− 1^ for ^90^Sr and 55.48 mGy∙h^− 1^∙MBq^− 1^ for ^90^Y (69.24 mGy∙h^− 1^∙MBq^− 1^ for the whole decay chain) shows a difference of just 3.4% from the S_Blood←Blood_ value, using the IDAC-Dose value as a reference.

The minimum S-value was observed for ^63^Ni (S_Lymph←Blood_ = 0.91 ± 0.14 mGy∙h^− 1^∙MBq^− 1^, S_Blood←Blood_ = 1.2555 ± 2E-5 mGy∙h^− 1^∙MBq^− 1^) and ^125^I (S_Lymph←Blood_ = 1.06 ± 0.15 mGy∙h^− 1^∙MBq^− 1^, S_Blood←Blood_ = 1.9260 ± 1E-4 mGy∙h^− 1^∙MBq^− 1^), with a -2.1% difference when compared with IDAC-Dose for ^125^I. All S-values are listed in Table 3 of the supplementary data. The relative error between S_Blood←Blood_ and S_Lymph←Blood_ ranged from − 44.8% for ^125^I to 0.7% for ^131^I (see supplemental Fig. 1).

### Alpha-emitting radionuclides

#### Alpha track quantification

**Fig. 3:**
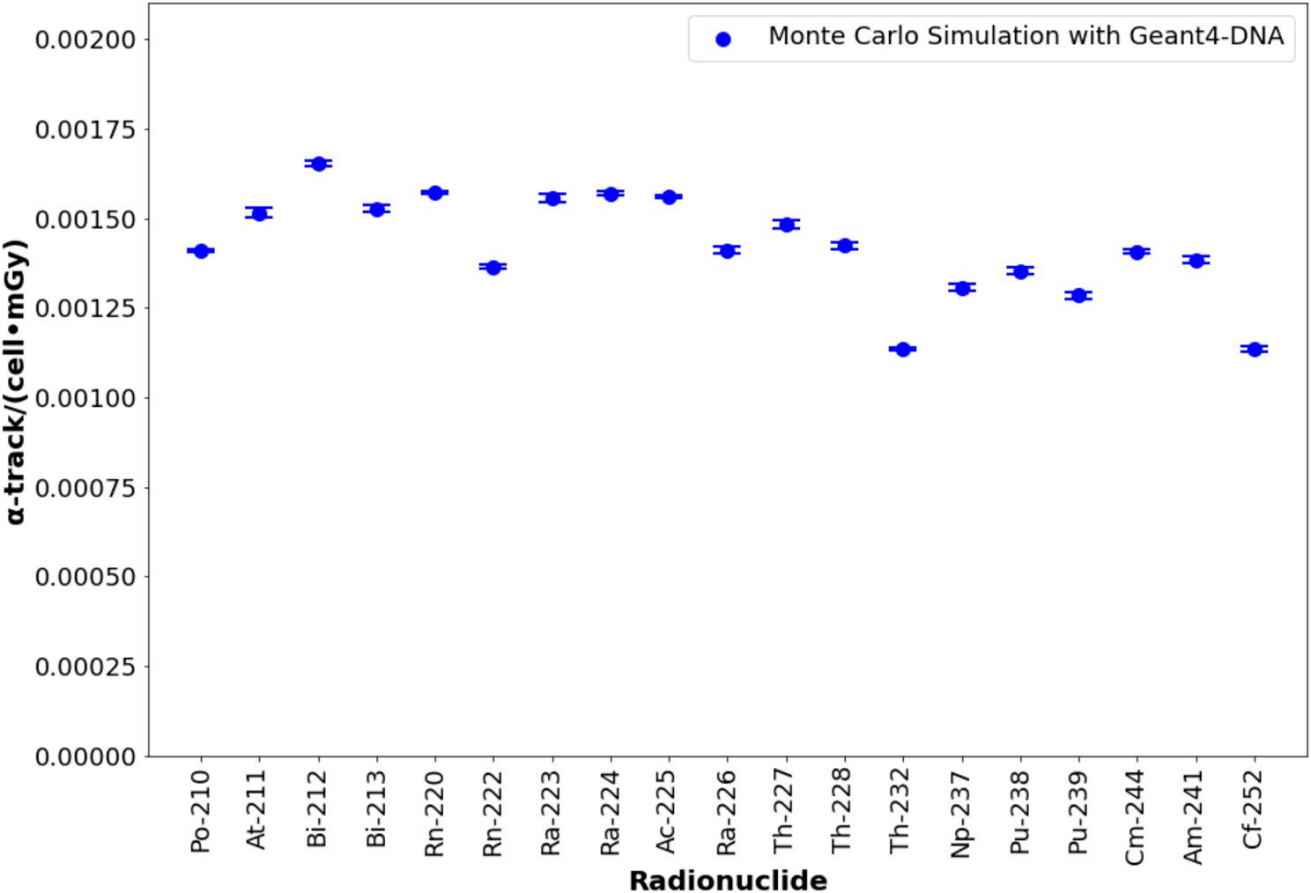
Number of alpha tracks per cell per mGy for alpha-emitting radionuclides. Error bars consider a coverage factor (k) of 2. ^223^Ra and ^225^Ac were previously studied and validated in [12]

**Fig. 4:**
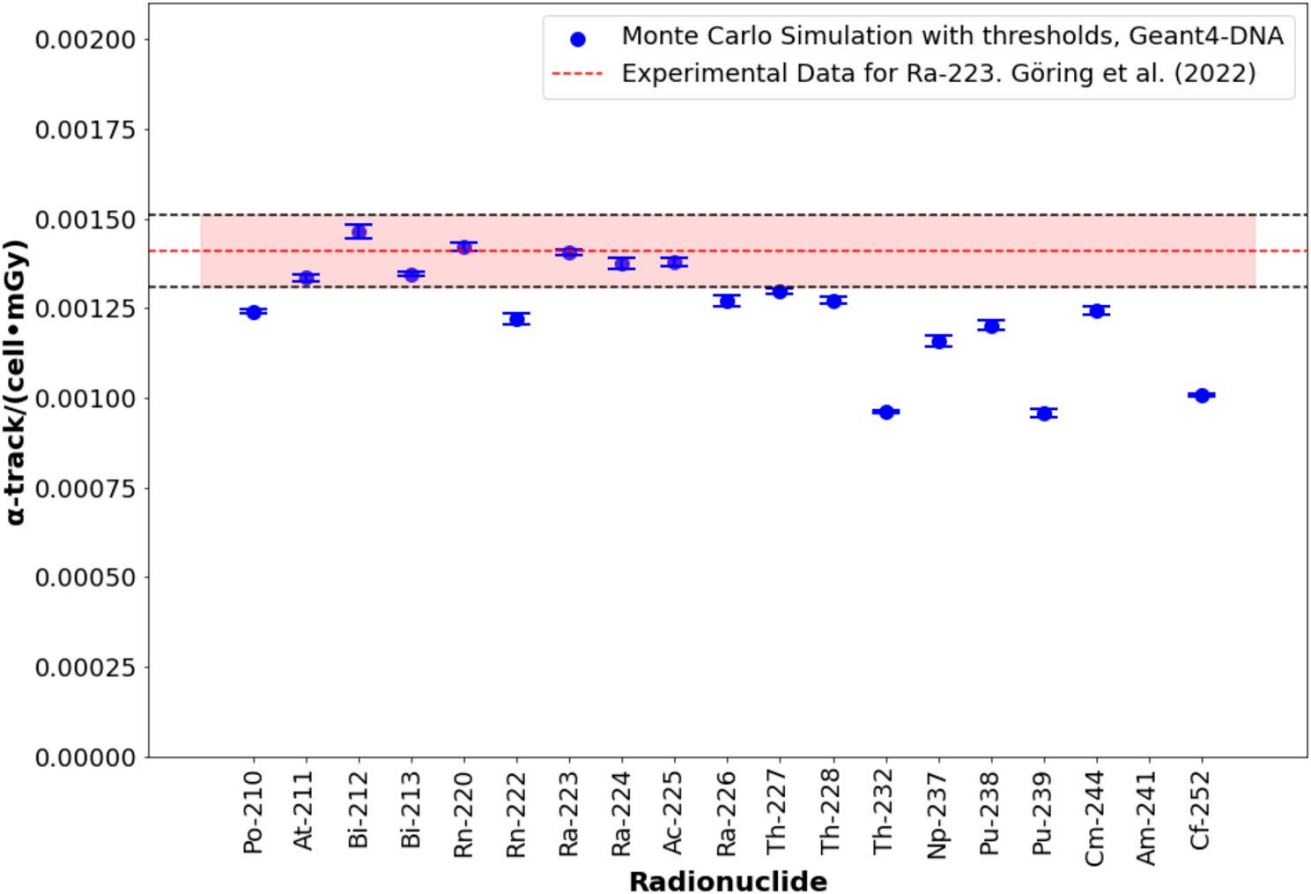
Number of alpha tracks per cell per mGy for alpha-emitting radionuclides after applying a threshold based on Ra-223 experimental data. Error bars consider a coverage factor (k) of 2. ^223^Ra and ^225^Ac were previously studied and validated in [12]

The number of α-tracks per cell per mGy ranged from 0.001134 ± 0.000008 α-tracks·cell⁻¹·mGy⁻¹ for ^252^Cf to 0.001654 ± 0.000008 α-tracks·cell⁻¹·mGy⁻¹ for ^212^Bi, as shown in Fig. 3 (numerical results are detailed in supplemental Table 4). After applying a selection threshold (track length > 0.75 μm and > 7 DSBs) based on ^223^Ra experimental data [4], the number ranged from 0.000960 ± 0.000003 α-tracks·cell⁻¹·mGy⁻¹ for ^252^Th to 0.001465 ± 0.000019 α-tracks·cell⁻¹·mGy⁻¹ for ^212^Bi, shown in Fig. 4.

#### DSBs quantification

**Fig. 5:**
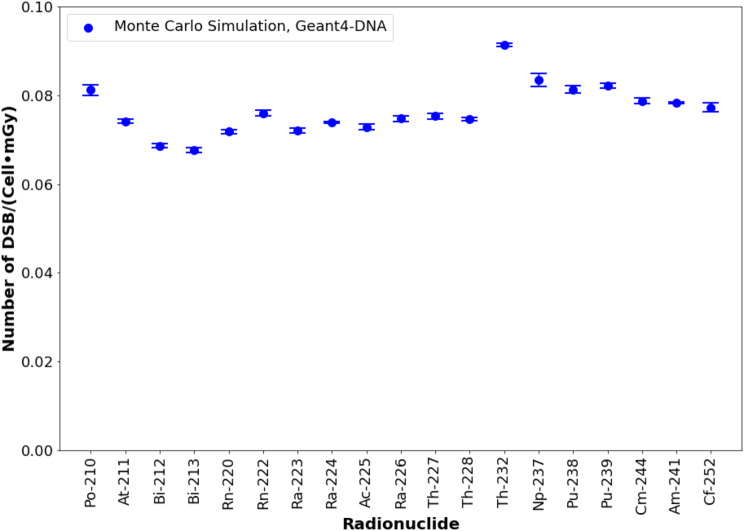
Number of DSBs per cell per mGy for alpha-emitting radionuclides. Error bars consider a coverage factor (k) of 2. ^223^Ra and ^225^Ac were previously studied and validated in [12]

The number of DSB per cell per mGy for alpha emitters ranged from 0.0677 ± 0.0005 DSB·cell⁻¹·mGy⁻¹ for ^213^Bi to 0.0914 ± 0.0004 DSBs·cell⁻¹·mGy⁻¹for ^232^Th, as shown in Fig. 5. Among radionuclides considered for therapeutic applications [34], ^227^Th shows the highest DNA damage with 0.0754 ± 0.0006 DSB·cell⁻¹·mGy⁻¹. For other radionuclides explored for therapeutic applications (^211^At, ^212^Bi, ^213^Bi, ^223^Ra, ^224^Ra, and ^225^Ac), the number of DSBs fell within a similar range, with a mean value of 0.0714 ± 0.0028 DSB·cell⁻¹·mGy⁻¹. Supplemental Table 5 provides the detailed numerical data corresponding to these results.

#### DSBs per micrometer of alpha track

**Fig. 6:**
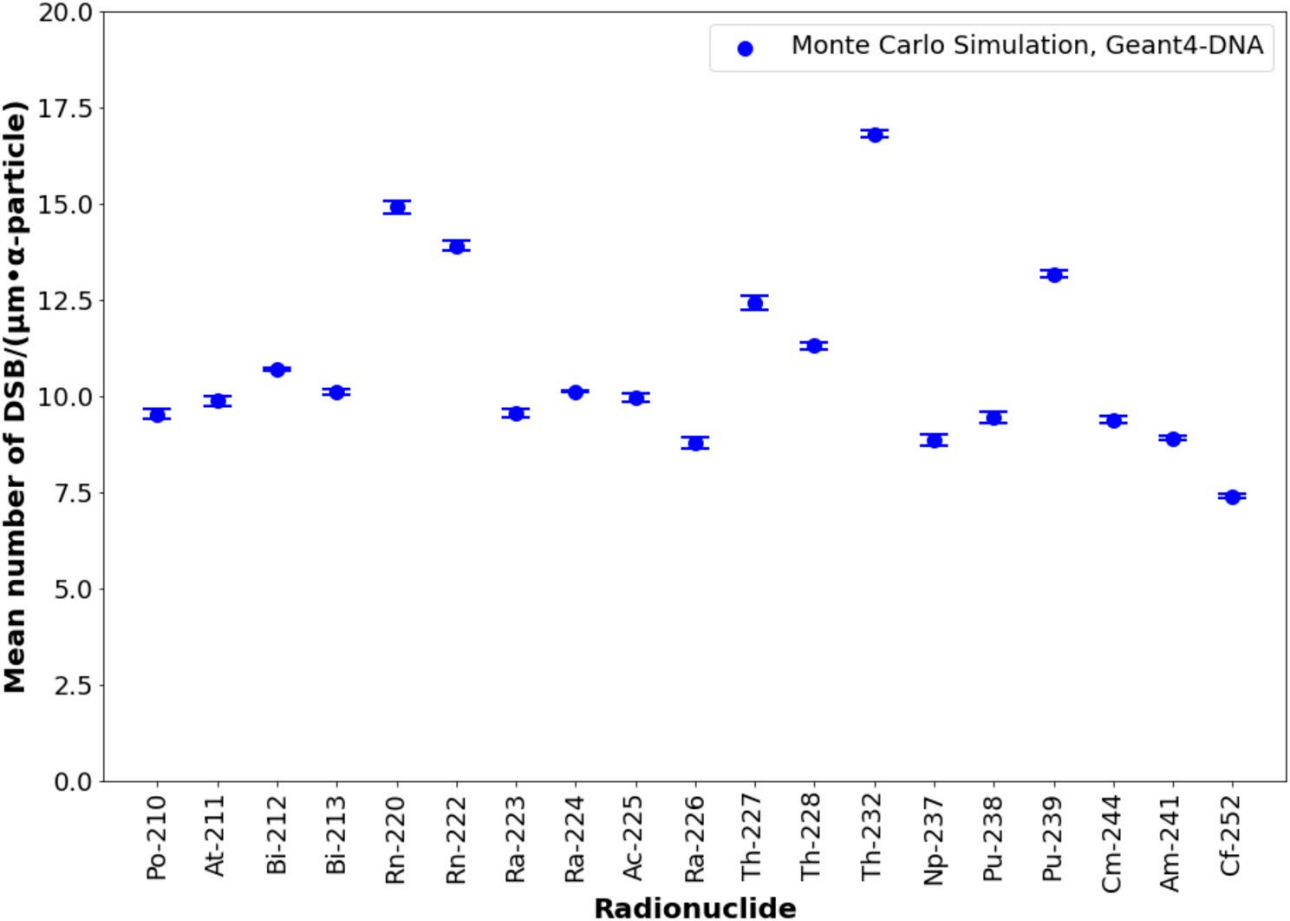
DNA double-strand break linear density (DSBs per micrometer) along alpha particle tracks for alpha-emitting radionuclides. Error bars consider a coverage factor (k) of 2. ^223^Ra and ^225^Ac were previously studied and validated in [12]

The DSB linear density, presented in Fig. 6, ranged from 7.4 ± 0.1 DSBs/µm for ^252^Cf to 16.8 ± 0.1 DSBs/µm for ^232^Th. There was strong agreement between the experimental value for ^223^Ra (9.5 DSBs/µm) [32] and the value obtained through Monte Carlo simulations (9.6 ± 0.1 DSBs/µm), as reported in our previous study [12]. For radionuclides with reported medical application (^211^At, ^212^Bi, ^213^Bi, ^223^Ra, ^224^Ra and ^225^Ac) [34, 35], the DSB linear density remained within a similar range, with a mean of 10.4 ± 1.0 DSBs/µm. Notably, ^227^Th (mean alpha particle energy of 5.9 MeV) and ^228^Th (mean alpha particle energy of 5.4 MeV), which are parents of ^223^Ra and ^224^Ra, respectively, exhibited higher DSB linear densities than their daughters: 12.4 ± 0.2 DSBs/µm for ^227^Th compared to 9.6 ± 0.1 DSBs/µm for ^223^Ra, and 11.3 ± 0.1 DSBs/µm for ^228^Th compared to 10.1 ± 0.02 DSBs/µm for ^224^Ra. The detailed numerical results are available in supplemental Table 6.

#### S-values

**Fig. 7:**
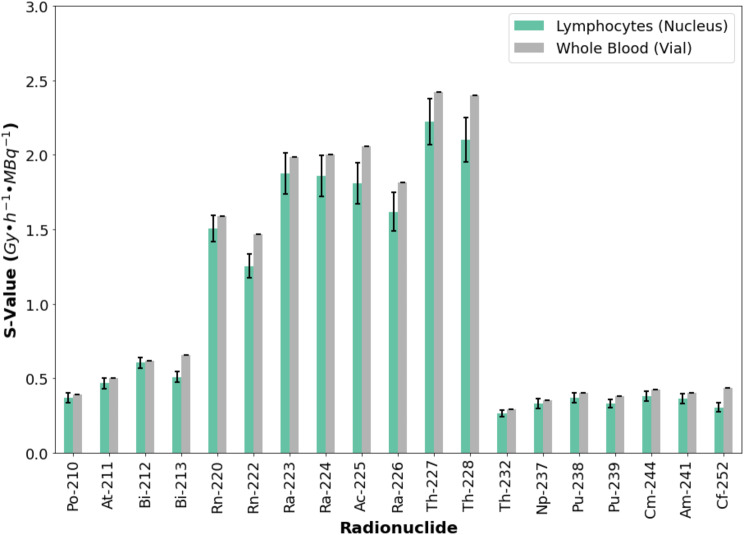
S-values for alpha-emitting radionuclides: Comparison between lymphocyte nuclei (S_Lymph←Blood_) and whole blood (S_Blood←Blood_). Error bars consider a coverage factor (k) of 2. S_Lymph←Blood_ considers only the contribution of alpha particles

The S-values for alpha emitters showed significant variation, with ^227^Th having the highest S-values (S_Lymph←Blood_ = 2.22 ± 0.16 Gy∙h^−^¹∙MBq⁻¹ and S_Blood←Blood_ = 2.42 ± 3E-5 Gy∙h⁻¹∙MBq⁻¹). The smallest value was observed for ^232^Th (S_Lymph←Blood_ = 0.26 ± 0.02 Gy∙h^−^¹∙MBq⁻¹ and S_Blood←Blood_ = 0.29 ± 2E-7 Gy∙h⁻¹∙MBq⁻¹). All S-values are listed in Table 7 of supplementary data. Additionally, the S-values for alpha emitters appeared proportional to the length of the radionuclide decay chain, as illustrated in Fig. 7. The relative error between S_Blood←Blood_ and S_Lymph←Blood_ ranged from − 29.8% for ^252^Cf to -2.1% for ^212^Bi (see supplemental Fig. 2).

Among the radionuclides studied, ^252^Cf was the only one that registered 21,069 neutrons and 12 protons in the phase-space from 4 × 10^9^ simulated nuclear transitions. Furthermore, ^222^Ra registered 2 protons in the phase-space from 2 × 10^9^ simulated nuclear transitions. For the calculation of S-values at the cellular level, only the energy deposited by alpha particles was considered.

## Discussion

The study highlights a clear differentiation in the biological effects between beta- and gamma-emitting radionuclides and alpha-emitting radionuclides.

### Beta- and Gamma-emitting radionuclides

Beta emitters such as ^32^P, ^89^Sr, ^90^Sr, ^188^Re, and ^228^Ra, with high-energy beta emissions, showed a similar number of DSBs per cell per mGy as radionuclides that are predominantly gamma emitters, such as ^99m^Tc and ^60^Co, or beta emitters with low-energy beta emissions (Fig. 1). However, the larger standard deviations (Fig. 1) observed for ^99m^Tc and ^75^Se (low-energy gamma emitters), and ^35^S and ^33^P (low-energy beta emitters), indicate large deposited energy fluctuations in their interactions with the cell nucleus. Furthermore, photons emitted by gamma emitters may escape from the radioactive solution volume. Consequently, the probability of interaction with the lymphocyte spheres is reduced in comparison to that of beta emitters, resulting in large fluctuations in the deposited energy and in the number of DSBs. In this study, to compensate this low interaction probability a large number of nuclear transitions (e.g., 8 × 10^9^ particles for ^99m^Tc and ^75^Se) was required to reach the appropriate deposited energy uncertainty (< 5%) in the cell nucleus in comparison with beta emitters (e.g., 4 × 10^9^ particles for ^35^S or ^89^Sr), which deposit their energy locally, or high energy gamma emitters (e.g., 4 × 10^9^ particles for ^60^Co or ^137^Cs), which may transfer a larger amount of energy to electrons inside the radioactive solution volume.

In this study as in our previous study [36], the calculation of DSBs per cell per mGy using Monte Carlo simulations follows the methodology used in irradiation experiments [3], where the number of DSBs are normalized by the absorbed dose in whole blood rather than the cell nucleus. ^125^I and ^63^Ni, both low-energy emitters, exhibited the lowest DSB induction values. ^125^I emits 35 keV gamma rays and internal conversion electrons (in the order of 20 keV to 30 keV) [25], while ^63^Ni primarily emits 17 keV beta particles. The low energy of these emissions results in a heterogeneous spatial distribution of energy, with fewer particles reaching the lymphocyte spheres, leading to reduced DNA damage and again large fluctuations in the deposited energy and number of DSB. For ^125^I and ^63^Ni, the absorbed dose in whole blood exceeds that in the cell nucleus, as reflected in their S-values (Fig. 2) and the large relative error between whole blood and cell nucleus S-values (supplemental Fig. 1). Consequently, the discrepancy between the S-values of whole blood and cell nucleus for ^35^S, ^63^Ni, ^75^Se, ^123^I, and ^125^I may be attributed to a heterogeneous distribution of the deposited energy at the microscopic level.

### Alpha-emitting radionuclides

Alpha particles, characterized by their high LET, produce dense ionization tracks, depositing energy over very short distances [37]. Supplemental Fig. 3 illustrates the correlation between the mean energy of the emitted alpha particle and the half-life of the parent radionuclide (data from ICRP Report 107 [38]). This study found that differences in the half-life of the daughters and the length of the decay chain across radionuclides significantly influence the number of induced DSBs at the same absorbed dose due to particle track length and the inverse correlation between particle energy and LET.

As shown in Fig. 3, the number of alpha tracks is approximately 0.0015 α-tracks per cell per mGy for most radionuclides, with the exception of ^210^Po and actinides (long half-lived radionuclides). In this study, the decay chains of these long half-life radionuclides were reduced to a single disintegration (emission of a single alpha) as part of the radionuclide selection criteria. Parent radionuclides with long half-lives produce alpha particles with lower mean energy compared to parents with shorter half-lives and faster-decaying daughters [39], thus reducing the probability for the alpha particle to reach the lymphocyte nuclei. Medically relevant radionuclides such as ^211^At, ^212^Bi, ^213^Bi, ^223^Ra, ^224^Ra, and ^225^Ac [34, 35] have short half-life parents and daughters that emit high-energy alpha particles. For example, considering only alpha decay, ^212^Bi (half-life: 60.5 min) emits alpha particles with a mean energy of 6.207 MeV (intensity: 35.93%). The remaining 64.07% decays through ^212^Po (half-life: 300 ns), which emits even higher-energy alpha particles (8.954 MeV, intensity: 100%). In contrast, ^232^Th (half-life: 14 × 10⁹ years) and ^252^Cf (half-life: 2.6 years) emit alpha particles with a mean energy of 4.081 MeV (intensity: 100%) and 6.219 MeV (intensity: 96.91%), respectively (radionuclide data from [40]). These differences in emitted alpha-particle energy may explain the observed variations in the number of alpha tracks per cell per mGy, where ^212^Bi exhibits the highest values, and ^252^Cf and ^232^Th the lowest. In the case of ^252^Cf, the emission probability of alpha particles (96.91%) may also reduce the number of alpha particles that reach the nucleus.

However, when analyzing the number of DSBs per cell per mGy (Fig. 5), ^210^Po and actinides ranked slightly higher than those with short half-lives (e.g., ^211^At, ^213^Bi, ^227^Th). This outcome can be attributed to the low mean energy of alpha particles, which results in higher LET and, consequently, greater energy deposition (i.e., a higher number of DSBs) by radionuclides such as ^210^Po, ^232^Th, or ^237^Np compared to ^212^Bi, ^213^Bi, or ^223^Ra.

The mean alpha track length per particle (supplemental Fig. 4) shows that radionuclides with long half-lives have slightly shorter track lengths compared to those with short half-lifes. ^232^Th shows the shortest track length, indicating that many alpha particles terminate their tracks within the cell nucleus. This suggests that these particles reach their Bragg peak within the nucleus, depositing a large amount of energy in this volume. This observation is reinforced by the data on DSB linear density in Fig. 7, where ^232^Th shows the highest value. The combination of DSB linear density with mean alpha track length explains why ^232^Th has the highest DSBs per cell per mGy.

For alpha particles, most experimental data comes from ^223^Ra studies [4, 32]. Figure 4 shows the number of alpha tracks per cell per mGy, after applying an experimentally derived threshold that aligns the simulation data with experimental observations for ^223^Ra. Experimental quantification of alpha tracks is inherently limited by the imaging resolution and the two-dimensional nature of microscopy-based image analysis. As a result, Monte Carlo simulations may quantify a higher number of alpha tracks than experimental data. Experimentally, the definition of an alpha track requires at least three adjacent DSB foci, corresponding to a minimum track length of 0.75–0.9 μm [32]. In addition, microscopy-based detection is biased towards alpha tracks that are approximately parallel to the imaging plane, potentially underestimating the true number of tracks. To account for these factors, thresholds validated in our previous study [36] (restricting alpha tracks to those longer than 0.75 μm and containing at least seven DSBs) resulted in good agreement between Monte Carlo predictions and experimental data for radionuclides with short half-lifes (e.g., ^211^At, ^212^Bi, ^224^Ra). However, radionuclides with truncated decay chains and long half-lives exhibit greater discrepancies, likely due to differences in their mean alpha particle energy and the probability of the alpha particle to reach the cell nucleus. The use of experimental thresholds provides a more realistic comparison, while indicates a potential experimental underestimation.

When analyzing the DSB linear density for alpha particles (Fig. 6), a strong agreement was found between experimental and Monte Carlo-based values, consistent with our previous study [36]. A similar observation is made when comparing the number of DSBs per cell per mGy with other Monte Carlo simulations. For example, Chatzipapas et al. [31] simulated the irradiation of human cells with a helium ion beam (external irradiation) at different energies. The study by Chatzipapas et al. differs from this one in terms of irradiation geometry and particle quality — external irradiation with a uniform helium ion beam versus internal irradiation with a broad alpha particle energy distribution — but their reported values for DSBs per cell per mGy are in a similar range to those found in this study for lymphocytes. Specifically, they calculated 0.065 DSB·cell^− 1^·mGy^− 1^ and 0.064 DSB·cell^− 1^·mGy^− 1^ for 5 MeV and 7.5 MeV helium ions, respectively, in lung carcinoma HTB-177 cells, and 0.071 DSB·cell^− 1^·mGy^− 1^ and 0.065 DSB·cell^− 1^·mGy^− 1^ for breast adenocarcinoma MCF-7 cells. These values are comparable to those found in this study for lymphocytes, ranging from 0.0686 ± 0.0004 DSB·cell^− 1^·mGy^− 1^ for ^212^Bi to 0.0914 ± 0.0004 DSB·cell^− 1^·mGy^− 1^ for ^232^Th. In the case of ^232^Th, the mean alpha energy is below the 5 MeV used by Chatzipapas et al..

Based on our discussion, we conclude that DSB linear density is a radionuclide-specific parameter. However, for radionuclides with reported medical applications (^211^At, ^212^Bi, ^213^Bi, ^223^Ra, ^224^Ra, and ^225^Ac) [34, 35], the DSB linear density is relatively similar (mean value: 10.4 ± 1.0 DSB/µm). A similar pattern is observed with the number of DSBs per cell per mGy (mean value: 0.071 ± 0.003 DSB·cell^− 1^·mGy^− 1^). The results for ^227^Th are notably higher for both parameters compared to other therapeutically relevant radionuclides (12.4 ± 0.2 DSB/µm and 0.0754 ± 0.003 DSB·cell^− 1^·mGy^− 1^), indicating that in the case of ^227^Th, there is an increase in the number of induced DSBs.

The inverse correlation between half-life and mean alpha particle energy [39, 41] (see supplemental Fig. 3) suggests that radionuclides with long half-life— many of which are produced in nuclear power plants and research reactors — may cause greater DNA damage due to their lower mean energy and higher LET, compared to alpha emitters with long decay chains and short half-life daughters, when cells are irradiated with the same absorbed dose.

### S-values

For all radionuclides, except ^131^I, the S-values for whole blood were larger than those for the lymphocyte nucleus. These differences are expected because our simulation model defines the cell as a cold source (without self-irradiation). The Geant4-DNA simulation allows the adjustment of the deposited energy through the low energy limit for secondary electron production (G4ProductionCutsTable thresholds). In this study, the threshold was optimized to minimize the difference between the S-values for the lymphocyte nucleus (S_Lymph←Blood_) and whole blood (S_Blood←Blood_), favoring a negative difference. The distribution of deposited energy at the microscopic level may explain the differences observed in low-energy gamma and beta emitters.

The consistency of the S-values for ^90^Sr and ^125^I with the sphere models of IDAC-Dose further validates the accuracy of the Monte Carlo simulations performed in this study. This comprehensive database of S-values for the vial geometry may be valuable for future experimental studies aimed at quantifying radiation-induced double-strand breaks in lymphocytes under internal ex vivo irradiation.

For alpha emitters, the largest differences were observed for ^252^Cf, which decays by spontaneous fission, generating neutrons that lead to proton production, potentially explaining the discrepancy between S_Lymph←Blood_ and S_Blood←Blood_. In the case of ^222^Rn (-14.5% relative error), the error may related with the number of pure beta emitting daughters (^214^Pb, ^214^Bi, ^210^Tl) which may lead to large beta contribution in S_Blood←Blood_ that is not considered in S_Lymph←Blood_ (only alpha particles are considered). The same may happen with ^226^Ra and ^228^Th.

Additionally, a confirmatory finding of this study is the strong correlation between the S-values of alpha-emitting radionuclides and the length of their decay chains (Fig. 7). Radionuclides with long decay chains, such as ²²⁵Ac, ²²⁷Th, and ²²⁸Th, exhibit higher S-values due to cumulative energy deposition from multiple alpha emissions. However, the extent of this accumulation depends on decay kinetics. Parents with short half-lives and rapidly decaying daughters can reach secular equilibrium within hours (e.g., ^223^Ra after 5 h: ^219^Rn, ^215^Po, ^211^Pb, ^211^Bi reach a ration A_daughter_/A_parent_ of 1, radionuclide data from [38, 40]), allowing both parent and progeny to contribute to irradiation. In contrast, long half-life parents may require years (e.g., ^232^Th after 5 h the largest contribution comes from ^228^Ra and ^228^Ac with a A_daughter_/A_parent_ ratio of 8.3 × 10^− 5^ and 2.254 × 10^− 5^, respectively, all other daughters were below 1 × 10^− 11^, radionuclide data from [38, 40]) to reach secular equilibrium, meaning that their decay contribution in ex vivo experiments (1 h irradiation) dominates as their daughters do not accumulate significantly. Consequently, short half-life parents with fast decaying daughters result in increased energy deposition from all members of the decay chain while long half-life parents contribute mainly through their own decay.

### Study limitations and future work

Positron-emitting radionuclides (e.g., ¹⁸F, ⁶⁸Ga, ^124^I) were not included due to the lack of positron interaction physics processes in Geant4-DNA. The implementation of these processes would require modifications to the Geant4 software, which exceeds the objectives and technical capabilities of this study.

This study focused primarily on DNA damage induced by beta, gamma, and alpha emissions, without separately quantifying the contribution of Auger and internal conversion electrons from radionuclides. Future investigations should evaluate the impact of these emissions on DNA damage. In addition, further studies are needed to evaluate the DNA damage caused by beta particles emitted in the decay chains of alpha-emitting radionuclides. For radionuclides with neutron emissions, further investigations are required to assess neutron-induced DNA damage. However, both experimental and simulated data on neutron-induced DNA damage remain scarce. Furthermore, Geant4-DNA currently lacks dedicated physics processes for neutron interactions, which limits its applicability for such studies.

This study focuses on the induction of DNA damage without incorporating DNA repair models. While the integration of a repair model is beyond the current scope, future work is planned to correlate DNA damage complexity (e.g., cluster size) and repair probability in combination with experimental data. In addition, this study investigates lymphocyte irradiation with unlabeled radionuclides, which provides an effective approach to understanding DNA damage and simulating models for radiation-induced DNA damage and repair. Furthermore, the Monte Carlo simulation model, based on the average lymphocyte cell and nucleus size, has shown good agreement with experimental data. However, further studies are needed to assess the sensitivity of the results to variations in cell and nucleus dimensions and geometry.

A potential improvement for future studies is to use the 3D alpha track information from Monte Carlo simulations to compute its planar projection in a 2D space, mimicking experimental imaging constraints. This approach could improve the agreement between simulations and experiments by accounting for track shortening due to projection effects and the resolution limitations of microscopic detection.

## Conclusion

This study investigates differences in the DNA damage induced by beta/gamma- and alpha-emitting radionuclides in lymphocyte nuclei. High-energy beta emitters induce DSBs similarly to gamma emitters, whereas low-energy emitters, show greater fluctuations due to the heterogeneous spatial distribution of deposited energy and variable interaction probabilities at the cellular level were observed. The spatial distribution of energy deposition at the microscopic level within the blood vial may be the most important factor explaining the fluctuations in the number of DSBs per cell per mGy.

In ex vivo irradiation, radionuclides with short half-lives and rapidly decaying daughters cumulatively contribute to energy deposition, resulting in high S-values due to the simultaneous presence of parent and progeny during irradiation. The number of DSBs induced is influenced by the average energy of the alpha emitters, with higher LET at lower alpha energies causing greater DNA damage. The results of this study suggest that long half-life radionuclides may cause more extensive DNA damage due to their higher LET. In addition, this work provides a comprehensive S-value database for future experimental studies of radiation-induced DNA damage in lymphocytes.

## Electronic supplementary material

Below is the link to the electronic supplementary material.

Supplementary Material 1

## Data Availability

All relevant data presented in this study are included in the article and supplementary materials. Further inquiries can be directed to the corresponding author.
