# Supplementary material for: In silico analysis of radiation-induced double-strand breaks by internal ex vivo irradiation of lymphocytes for 45 alpha- and beta/gamma-emitting radionuclides
Source: EJNMMI Res. 2025 Mar 10;15:21. doi: 10.1186/s13550-025-01214-w (PMC11893945; doi:10.1186/s13550-025-01214-w)
Supplement: Supplementary file 1 — Supplementary Material 1 [file 13550_2025_1214_MOESM1_ESM.pdf]

In Silico Analysis of Radiation-Induced Double-Strand Breaks by Internal Ex Vivo Irradiation of Lymphocytes for 45 Alpha- And Beta/Gamma-Emitting Radionuclides

Author Names and Degrees:

Dr. Maikol Salas-Ramirez[1]*,

Prof. Dr. Michael Lassmann[1],

PD Dr. Uta Eberlein[1]

Affiliations:

[1]Department of Nuclear Medicine, University Hospital Würzburg, Würzburg, Germany

***Contact information:** Maikol Salas Ramirez, Department of Nuclear Medicine, University Hospital Würzburg, Oberdürrbacher Str. 6, 97080 Würzburg, Germany, E-Mail: E_Salas_M@ukw.de

SUPPLEMENTAL FIGURES

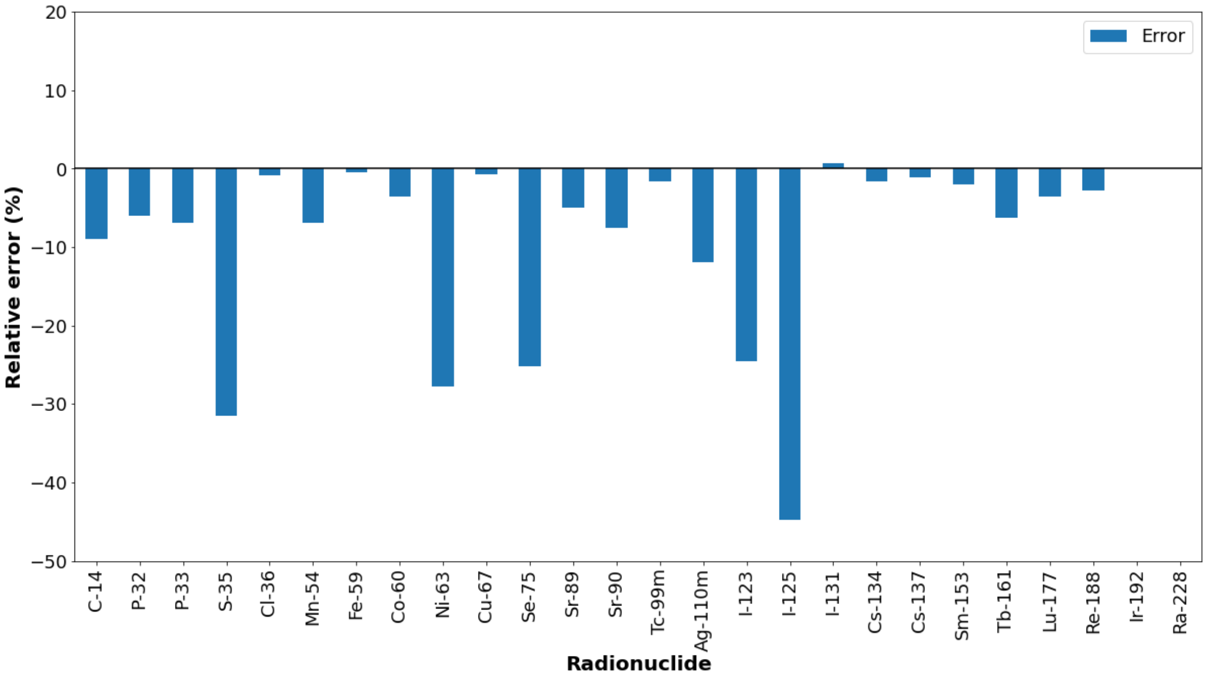

Supplemental figure 1. Relative error between S_{Lymph} and S_{Blood} (reference: S_{Blood}) for beta/gamma emitting radionuclides.

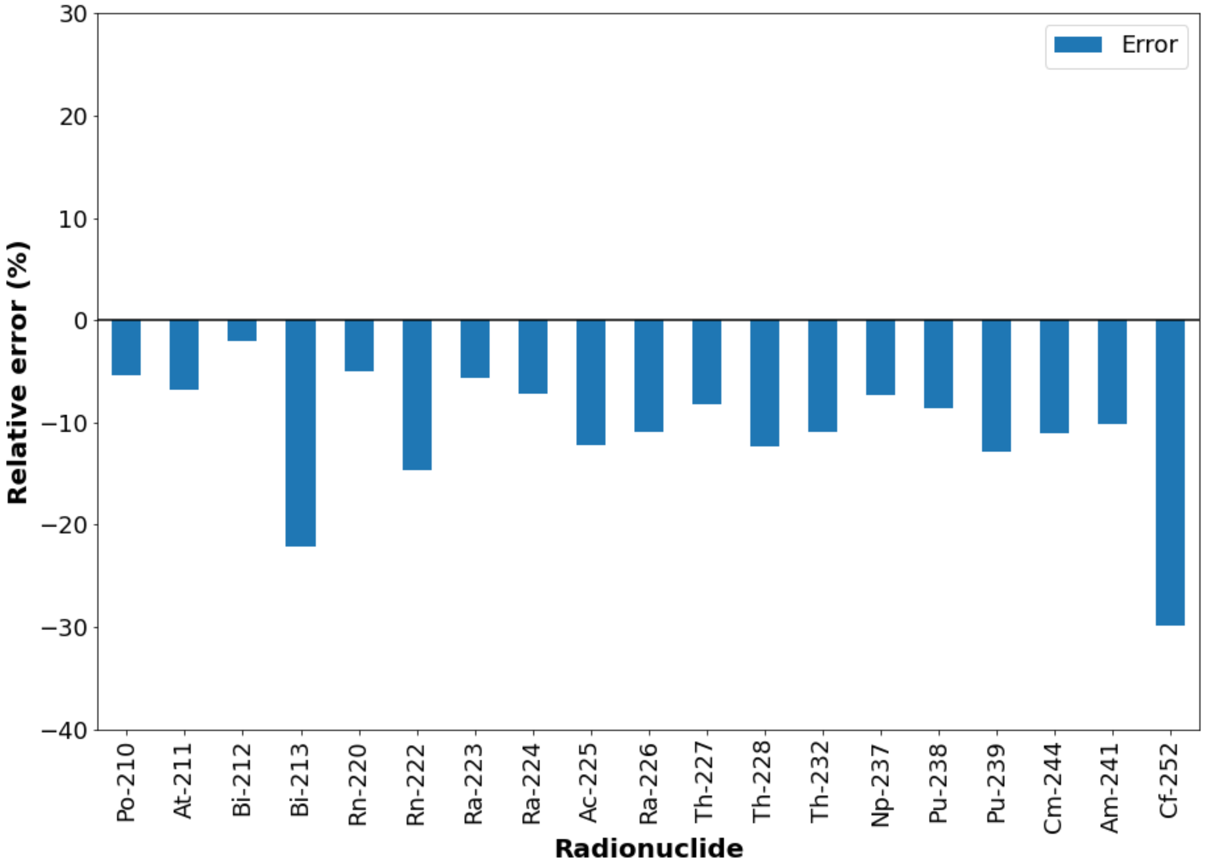

Supplemental figure 2. Relative error between S_{Lymph} and S_{Blood} (reference: S_{Blood}) for alpha emitting radionuclides.

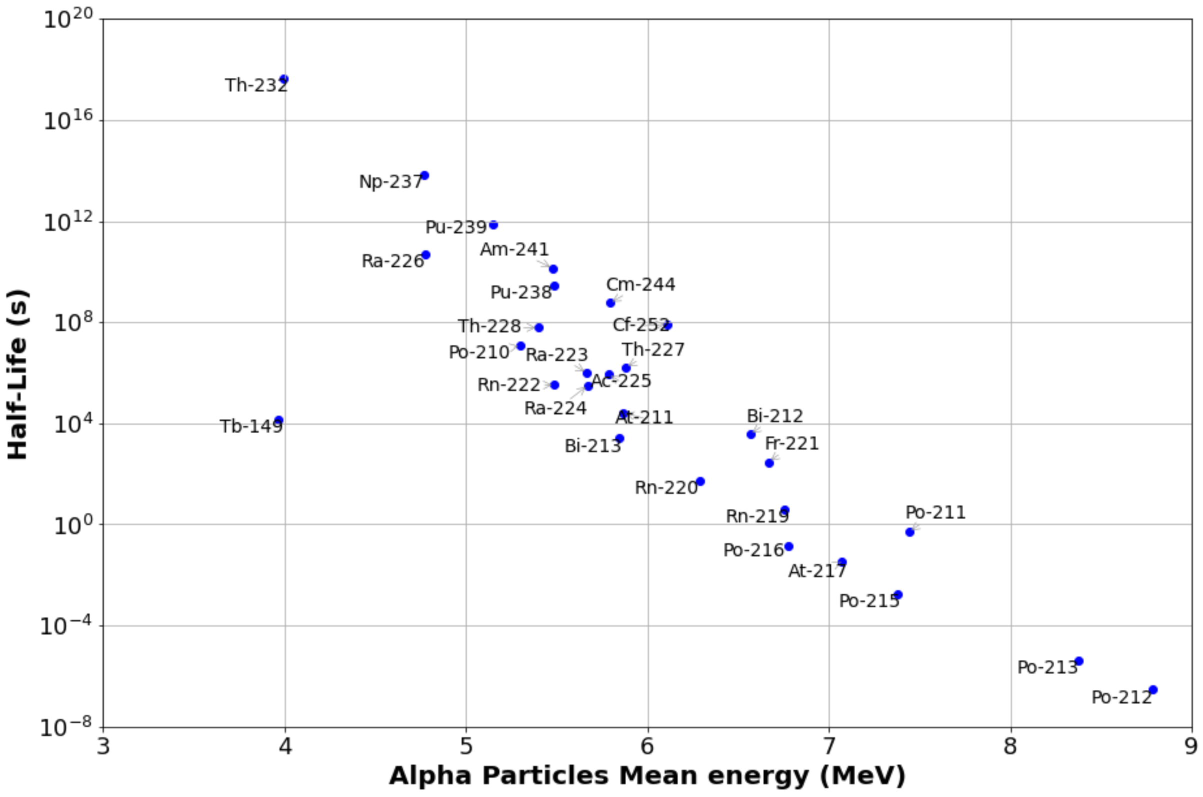

Supplemental figure 3. Correlation between mean alpha particle energy and radionuclide half-life.

Data sourced from ICRP Report 107 [1]

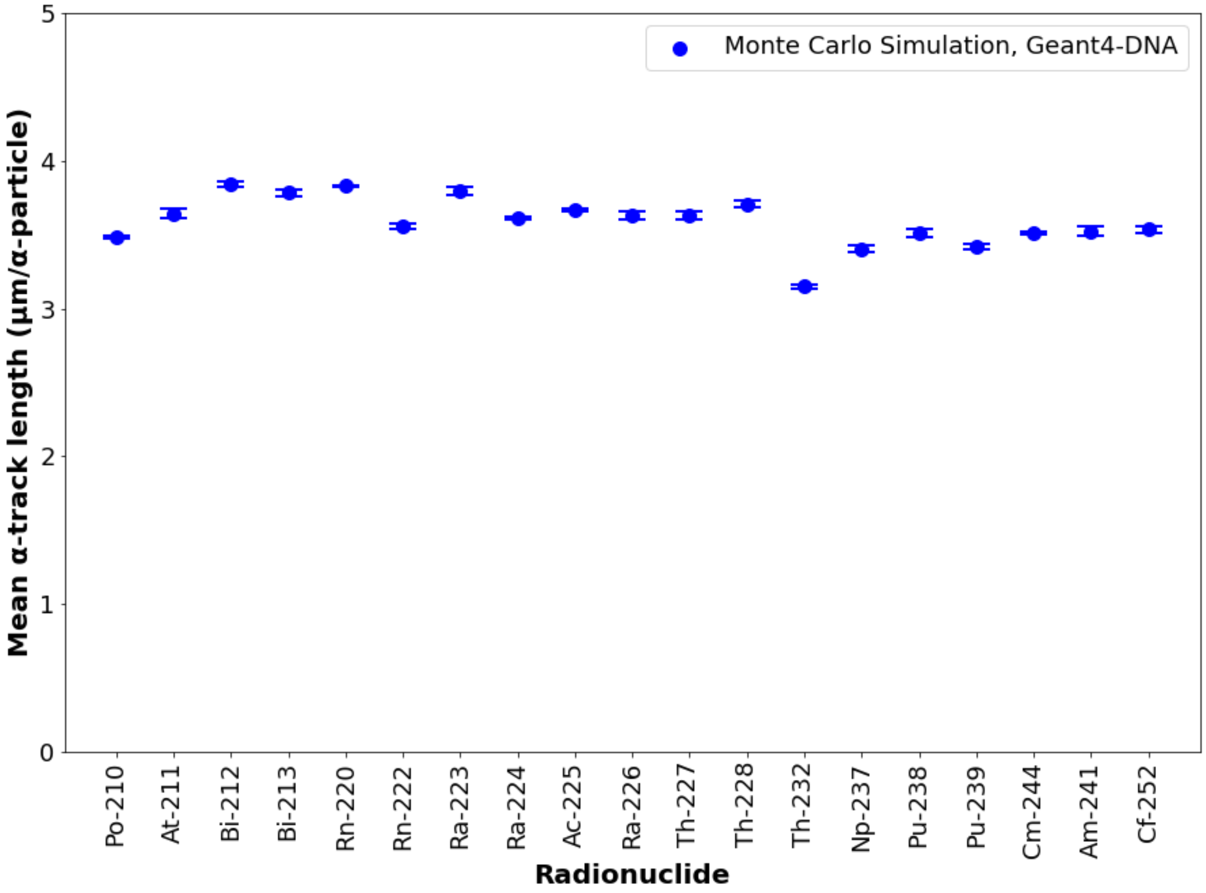

Supplemental figure 4. Average Track Length of Alpha Particles in Lymphocytes across Explored Alpha-Emitting Radionuclides.

SUPPLEMENTAL TABLES

Supplemental table 1. Number for simulated nuclear transitions in whole blood simulation (macroscopic simulation) for each radionuclide

Radionuclide	Number of nuclear transitions
Ac-225	1000000000
Am-241	4000000000
At-211	4000000000
Bi-212	4000000000
Bi-213	4000000000
C-14	4000000000
Cf-252	4000000000
Cl-36	4000000000
Cm-244	4000000000
Co-60	4000000000
Cs-134	4000000000
Cs-137	4000000000
Cu-67	4000000000
Fe-59	8000000000
I-123	8000000000
I-125	8000000000
I-131	4000000000
Ir-192	4000000000
Lu-177	4000000000
Mn-54	4000000000
Ni-63	8000000000
Np-237	4000000000
P-32	4000000000
P-33	4000000000
Po-210	4000000000
Pu-238	4000000000
Pu-239	6000000000
Ra-223	1000000000
Ra-224	1000000000
Ra-226	1000000000
Ra-228	4000000000
Re-188	4000000000
Rn-220	2000000000
Rn-222	2000000000

S-35	4000000000
Se-75	8000000000
Sm-153	4000000000
Sr-89	4000000000
Sr-90	4000000000
Tb-161	4000000000
Tc-99m	8000000000
Th-227	1000000000
Th-228	1000000000
Th-232	8000000000
Y-90	2000000000

Supplemental table 2. Number of DSB_{MC} obtained from the Monte Carlo simulation (Geant4-DNA) for beta- and gamma-emitters.

Radionuclide	$DSB_{MC}\left(\frac{DSB}{cell \cdot mGy}\right)$	
	Mean	SD
C-14	0.013	0.004
P-32	0.012	0.001
P-33	0.010	0.004
S-35	0.012	0.004
Cl-36	0.011	0.001
Mn-54	0.010	0.003
Fe-59	0.011	0.002
Co-60	0.012	0.001
Ni-63	0.009	0.004
Cu-67	0.012	0.003
Se-75	0.011	0.00
Sr-89	0.013	0.001
Sr-90	0.013	0.001
Y-90	0.014	0.002
Tc-99m	0.015	0.005
I-123	0.013	0.003
I-125	0.006	0.003
I-131	0.012	0.001
Cs-134	0.012	0.003
Cs-137	0.012	0.001
Sm-153	0.011	0.002
Tb-161	0.015	0.002

Lu-177	0.011	0.002
Re-188	0.013	0.001
Ir-192	0.011	0.002
Ra-228	0.012	0.001

SD: Standard Deviation, coverage factor (k) of 2

Supplemental table 3. S-Values for lymphocyte nuclei (S_{Lymph}) and whole blood (S_{Blood}) for gamma and beta emitters in a water medium with a density equal to 1g/cm^3.

Radionuclide	$S_{Lymph}\left(\frac{mGy}{MBq\cdot h}\right)$		$S_{Blood}\left(\frac{mGy}{MBq\cdot h}\right)$	
	Mean	SD (k=2)	Mean	SD (k=2)
C-14	3.24	0.32	3.5550	0.0002
P-32	40.21	0.91	42.8040	0.0011
P-33	5.08	0.38	5.4630	0.0002
S-35	2.40	0.26	3.5010	0.0002
Cl-36	16.85	0.58	17.0010	0.0005
Mn-54	1.66	0.17	1.7820	0.0004
Fe-59	10.00	0.33	10.0485	0.0005
Co-60	10.03	0.51	10.4040	0.0007
Ni-63	0.91	0.14	1.2555	0.0000
Cu-67	10.80	0.51	10.8810	0.0004
Se-75	1.53	0.15	2.0475	0.0002
Sr-89	35.14	0.84	36.9900	0.0011
Sr-90	61.83	1.17	66.9150	0.0016
Y-90	52.38	1.48	53.2440	0.0014
Tc-99m	1.24	0.13	1.4130	0.0001
I-123	2.14	0.18	2.8305	0.0002
I-125	1.06	0.15	1.9260	0.0001
I-131	14.31	0.55	14.2110	0.0005
Cs-134	13.81	0.55	14.0490	0.0005
Cs-137	17.81	0.61	18.0180	0.0007
Sm-153	18.56	0.65	18.9450	0.0005
Tb-161	12.61	0.57	13.4460	0.0004
Lu-177	10.15	0.48	10.5300	0.0004
Re-188	45.80	0.98	47.1150	0.0014
Ir-192	16.60	0.58	16.5960	0.0005
Ra-228	33.18	0.85	33.1560	0.0011

SD: Standard Deviation, coverage factor (k) of 2

Supplemental table 4. Number of α-tracks obtained from the Monte Carlo simulation (Geant4-DNA) for alpha emitters.

Radionuclide	$\alpha\text{-}tracks_{MC}\left(\frac{\alpha\text{-}track}{cell\cdot mGy}\right)$	
	Mean	SD (k=2)
Po-210	0.001408	0.000004
At-211	0.001515	0.000013
Bi-212	0.001654	0.000008
Bi-213	0.001527	0.000009
Rn-220	0.001570	0.000004
Rn-222	0.001364	0.000005
Ra-223	0.001555	0.000010
Th-227	0.001569	0.000005
Ra-224	0.001622	0.000010
Th-228	0.001411	0.000009
Ac-225	0.001559	0.000005
Ra-226	0.001424	0.000010
Th-232	0.001135	0.000005
Np-237	0.001306	0.000010
Pu-238	0.001353	0.000011
Pu-239	0.001284	0.000009
Cm-244	0.001406	0.000005
Am-241	0.001384	0.000011
Cf-252	0.001134	0.000008

SD: Standard Deviation, coverage factor (k) of 2

Supplemental table 5. Number of DSB_{MC} obtained from the Monte Carlo simulation (Geant4-DNA) for alpha emitters.

Radionuclide	$DSB_{MC}\left(\frac{DSB}{cell\cdot mGy}\right)$	
	Mean	SD (k=2)
Po-210	0.0812	0.0012
At-211	0.0742	0.0005
Bi-212	0.0686	0.0004
Bi-213	0.0677	0.0005
Rn-220	0.0721	0.0004
Rn-222	0.0718	0.0006
Ra-223	0.0721	0.0005
Th-227	0.0754	0.0006
Ra-224	0.0739	0.0002
Th-228	0.0746	0.0004

Ac-225	0.0729	0.0007
Ra-226	0.0748	0.0007
Th-232	0.0914	0.0004
Np-237	0.0835	0.0014
Pu-238	0.0813	0.0009
Pu-239	0.0823	0.0006
Cm-244	0.0788	0.0006
Am-241	0.0783	0.0002
Cf-252	0.0773	0.0010

SD: Standard Deviation, coverage factor (k) of 2

Supplemental table 6. Linear density of DSBs per micrometer along alpha tracks in lymphocyte nuclei for alpha emitters.

Radionuclide	$DSB_{MC}\left(\frac{DSB}{\mu m \cdot \alpha\text{-}Track}\right)$	
	Mean	SD (k=2)
Po-210	9.5	0.1
At-211	9.9	0.1
Bi-212	10.7	0.0
Bi-213	10.1	0.1
Rn-220	14.9	0.2
Rn-222	13.9	0.1
Ra-223	9.6	0.1
Th-227	12.4	0.2
Ra-224	10.1	0.0
Th-228	11.3	0.1
Ac-225	10.0	0.1
Ra-226	8.8	0.1
Th-232	16.8	0.1
Np-237	8.9	0.1
Pu-238	9.4	0.1
Pu-239	13.2	0.1
Cm-244	9.4	0.1
Am-241	8.9	0.1
Cf-252	7.4	0.1

SD: Standard Deviation, coverage factor (k) of 2

Supplemental table 7. -Values for lymphocyte nuclei (S_{Lymph}) and whole blood (S_{Blood}) for alpha emitters in a in a water medium with a density equal to 1g/cm^3.

Radionuclide	$S_{Lymph}\left(\frac{Gy}{MBq\cdot h}\right)$		$S_{Blood}\left(\frac{Gy}{MBq\cdot h}\right)$	
	Mean	SD (k=2)	Mean	SD (k=2)
Po-210	0.37	0.03	0.389151	0.000001
At-211	0.47	0.04	0.501975	0.000003
Bi-212	0.60	0.04	0.616473	0.000005
Bi-213	0.51	0.04	0.653076	0.000004
Rn-220	1.51	0.09	1.586376	0.000008
Rn-222	1.25	0.08	1.466856	0.000005
Ra-223	1.88	0.14	1.986768	0.000001
Th-227	2.22	0.16	2.002176	0.000008
Ra-224	1.86	0.14	2.059560	0.000005
Th-228	2.10	0.15	1.816956	0.000006
Ac-225	1.81	0.14	2.059128	0.000005
Ra-226	1.62	0.13	2.399220	0.000008
Th-232	0.26	0.02	0.2939040	0.0000004
Np-237	0.33	0.03	0.354708	0.000001
Pu-238	0.37	0.03	0.402417	0.000001
Pu-239	0.33	0.03	0.377430	0.000001
Cm-244	0.38	0.03	0.424539	0.000001
Am-241	0.36	0.03	0.403947	0.000001
Cf-252	0.31	0.03	0.434871	0.000005

SD: Standard Deviation, coverage factor (k) of 2

REFERENCES

1. Eckerman, K. and A. Endo, *ICRP Publication 107. Nuclear decay data for dosimetric calculations.* Ann ICRP, 2008. **38**(3): p. 7-96.
